# Upregulation of FAM134B inhibits endoplasmic reticulum stress‐related degradation protein expression and promotes hepatocellular carcinogenesis

**DOI:** 10.1111/jcmm.17964

**Published:** 2023-09-20

**Authors:** Houhong Wang, Lu Liu, Huihui Gong, Heng Li

**Affiliations:** ^1^ Department of General Surgery The Affiliated Bozhou Hospital of Anhui Medical University Bozhou China; ^2^ Department of Endocrine Department The Affiliated Nantong Hospital of Shanghai Jiao Tong University Nantong China; ^3^ Faculty of Health and Life Sciences Oxford Brookes University Oxford England UK; ^4^ Department of Comprehensive Surgery, Anhui Provincial Cancer Hospital West District of The First Affiliated Hospital of USTC Hefei China

**Keywords:** cell autophagy, endoplasmic reticulum stress, FAM134B, hepatocellular carcinoma

## Abstract

Endoplasmic reticulum (ER) stress can stimulate the proliferation and metastasis of hepatocellular carcinoma (HCC) cells while hindering apoptosis and immune system function, but the molecular mechanism of ER stress in HCC has yet to be fully studied. We aim to investigate the molecular mechanism by which FAM134B inhibits autophagy of HCC cells by reducing the expression of ER stress‐related degradation proteins. Clinical samples were collected for this study. Normal liver cell lines HL7702 and Hep3B and Huh7 HCC cell lines were cultured. Construction of FAM134B knockdown cell line. Cell proliferation was measured using the CCK‐8 assay, while cell migration and invasion capabilities were detected using the plate colony formation assay. Flow cytometry was used to detect the apoptosis rate. Transmission electron microscopy was used to observe the formation of autophagosomes. qRT‐PCR and WB detective expression changes related to autophagy proteins. Finally, the expression of the relevant proteins was observed by immunohistochemistry. The expression of FAM134B was significantly increased in human liver cancer tissue and HCC cell lines Hep3B and Huh7. After the lentiviral vector was transfected into Hep3B cells with sh‐FAM134B, results showed that sh‐FAM134B could effectively inhibit Hep3B cell proliferation and promote HCC cell apoptosis. Meanwhile, sh‐FAM134B could effectively induce the autophagy of Hep3B liver cancer cells. Immunohistochemistry results showed that sh‐FAM134B could effectively induce ER stress. FAM134B inhibits HCC cell autophagy and promotes the progression of liver cancer by inhibiting the expression of ER stress‐related degradation factors such as DERL2, EDEM1, SEL1L and HRD1.

## 1 INTRODUCTION

Global statistics indicate a continuous increase in the incidence and mortality rate of hepatocellular carcinoma (HCC).^1^ HCC is ranked as the fifth most prevalent cancer globally, accounting for approximately 7% of all cancers and resulting in an estimated 800,000 deaths annually.^2^, ^3^ Early detection and diagnosis of HCC are challenging due to its aggressive nature, resulting in a majority of patients to receive treatment at advanced stages. Numerous clinical studies have revealed that the occurrence and survival of HCC are linked to various risk factors such as hepatitis B and C virus infection, excessive alcohol consumption, obesity, diabetes and fatty liver disease.^4^, ^5^ Consequently, HCC poses a significant disease burden and presents a high mortality risk, necessitating increased focus on its prevention and treatment strategies.

The pathophysiological mechanisms of HCC remain intricate and incompletely elucidated. Current research primarily emphasizes the following areas: chronic inflammation resulting from prolonged hepatitis virus infection, alcohol abuse, obesity, fatty liver disease and other factors may induce cellular damage, cell death, liver fibrosis and other pathological alterations, thereby enhancing the development of HCC.^6^, ^7^ The occurrence of HCC is closely associated with gene mutations, including p53, β‐catenin and others. Distinct gene mutations can lead to diverse pathological changes, such as hepatocellular proliferation, invasion and metastasis.^8^ Hepatocytes sustain hepatic functional equilibrium through regeneration and apoptosis. Imbalances between hepatocyte regeneration and apoptosis can spur excessive hepatocarcinoma cell proliferation and disruption of normal cellular growth control, eventually precipitating HCC formation.^9^, ^10^ Considerable abnormalities in angiogenesis are observed in HCC. Neovascularization differentiation and expansion within cancerous tissue constitute important mechanisms for HCC invasion and metastasis, providing abundant nourishment and oxygen to HCC cells.^11^

Endoplasmic reticulum (ER) stress represents a physiological and pathological state wherein the ER encounters diverse internal or external pressures or injuries, culminating in ER dysfunction and abnormal protein aggregation, which elicits a cascade of biological reactions such as inflammatory responses and cell death.^12^, ^13^ Liver cell injury and transformation can arise due to ER stress. By activating multiple signalling pathways, ER stress undermines the liver cell growth inhibition pathway, promotes cell cycle progression and facilitates cell proliferation, thereby fostering the malignant transformation of liver cells.^14^ Additionally, ER stress can contribute to liver fibrosis and precancerous lesions. In conditions like chronic hepatitis, ER stress prompts the proliferation of mesenchymal cells (such as hepatic stellate cells), culminating in liver fibrosis. Simultaneously, ER stress activates a series of signalling pathways, establishing a conducive microenvironment for precancerous lesions and ultimately driving the development of HCC.^15^ Among the transcriptional response genes to ER stress, FAM134B can recruit ATF6 molecules into the ER to activate the ER stress response.^16^

However, the precise mechanism of FAM134B on the occurrence and progression of HCC mediated through the activation of the ER stress response remains incompletely understood. Therefore, this study aims to employ in vitro cellular experiments to investigate the molecular mechanism by which FAM134B inhibits the expression of ER stress‐related degradation proteins and facilitates the progression of HCC.

## 2 MATERIALS AND METHODS

### 2.1 Materials

Human liver cancer and adjacent normal tissue specimens were collected from patients who underwent surgical resection at the Affiliated Bozhou Hospital of Anhui Medical University (Ethics approval number: 20230113). The human normal liver cell line HL7702, and HCC cell lines Hep3B and Huh7 were procured from Wuhan Procell Life Sciences Co., Ltd. DMEM high‐glucose culture medium and fetal bovine serum were purchased from Thermo Fisher Scientific Inc. Trypsin, cell lysis buffer and SDS‐PAGE gel kit were sourced from Shanghai Beyotime Biotechnology Co., Ltd. The CCK‐8 assay kit was purchased from Hangzhou Vigor Biosciences Co., Ltd. The cell cryopreservation solution was purchased from China Newzyme Co., Ltd. Antibodies against GAPDH, FAM134B, LC3B, Beclin1, p62, DERL2, EDEM1, SEL1L and HRD1 were purchased from Abmart Biotechnology (Shanghai) Co., Ltd. The goat anti‐rabbit IgG secondary antibodies labelled with horseradish peroxidase were purchased from Beijing Zhongshan Jinqiao Biotechnology Co., Ltd. The FAM134B interference lentivirus was purchased from Shanghai Hanheng Biotechnology Co., Ltd.

### 2.2 Cell culture

The HL7702, Hep3B and Huh7 cell lines were cultured in DMEM supplemented with 10% fetal bovine serum within a controlled cell incubator. Subculturing of the cells was performed upon reaching approximately 90% confluence, in accordance with experimental requirements.

### 2.3 Lentiviral transduction

Recombinant lentiviral vectors targeting FAM134B and empty vectors were generated. Hep3B cells were transduced using a multiplicity of infection (MOI) ranging from 10 to 100, in the presence of 5 mg/mL polybrene. After transduction, the viral‐containing medium was replaced with fresh DMEM medium. Following a 72‐h incubation, cells were selected to with 2 μg/mL puromycin to establish a stable sh‐FAM134B cell line.

### 2.4 Cell proliferation assay

Cell viability was assessed using the CCK‐8 cell proliferation assay. Cells were seeded at a density of 2 × 104 cells/well in a 96‐well plate, containing 100 μL of complete DMEM high‐glucose culture medium, and incubated for 24 h. For Hep3B cells transduced with lentivirus, the assay was performed accordingly. Next, the supernatant was removed, and the cells were treated with 10 μL of CCK‐8 solution. The optical density value was measured at a wavelength of 450 nm.

### 2.5 Flat clone experiment

Log‐phase cells were harvested and dissociated into a single‐cell suspension using 0.25% trypsin. Subsequently, these cells were reseeded at a density of 500 cells/well onto a 6‐well plate. The plate was vigorously shaken and then incubated for 2 weeks to allow single cells to form visible cell clusters. Cells were washed twice with PBS, followed by fixation with 4% paraformaldehyde for 15 min. After additional rinses with PBS, the cells were stained with a solution of 3.7% methanol and 0.1% crystal violet for 30 min.

### 2.6 Flow cytometry for detecting cell apoptosis

Cell apoptosis was assessed using the V‐FITC/PI apoptosis detection kit (ThermoFisher Scientific). Initially, cells were washed with pre‐cooled PBS and collected at a density of 5 × 10^5^ cells. 1× Binding Buffer was prepared by diluting 5× Binding Buffer with double‐distilled water. The cells were then resuspended in 500 μL of the buffer. Annexin V‐FITC and PI were added to each tube, followed by gentle vortexing and a 5‐min incubation in the dark. Subsequently, the cells were analysed using a flow cytometer. The acquired data were subsequently analysed using FlowJo software.

### 2.7 Transmission electron microscopy for observing autophagosome formation

Hep3B liver cancer cells were seeded into a 6‐well plate. Subsequently, log‐phase cells were detached using 0.25% trypsin and fixed with 4% glutaraldehyde for 2 h at 4°C. Further fixation was performed using 1% osmium tetroxide for an additional 2 h. The cells were then stained with filtered uranyl acetate and dehydrated through a graded series of ethanol and acetone. Subsequently, the cells were embedded in Epon resin, and the semi‐thin (0.5 μm) and ultra‐thin (60 nm) sections were prepared. These sections were stained with lead citrate and uranyl acetate, followed by fixation with 2.5% glutaraldehyde and 1% osmium tetroxide, dehydration in progressively concentrated alcohol and final embedding in Epon resin. Visualization of the sections was accomplished using a Hitachi H‐7500 transmission electron microscope, and images were captured using a Gatan‐780 system.

### 2.8 qRT‐PCR

To extract total RNA, cells were lysed in a 1.5 mL EP tube with 1 mL of Trizol reagent for 10 min. Chloroform was subsequently added, and the mixture was centrifuged at 4°C and 6216 *g*. The upper aqueous phase was carefully transferred to a new tube and mixed with 400 μL of isopropanol. After several rounds of centrifugation, the supernatant was discarded, and the resulting pellet was dissolved in 20 μL DEPC water. Reverse transcription of RNA into cDNA was carried out at 25°C for 5 min, 50°C for 15 min, 85°C for 5 min and 4°C for 10 min. The cDNA was subsequently diluted 10‐fold for real‐time PCR amplification. The housekeeping gene GAPDH was used as the internal reference, and the primer sequences are provided in Table 1.

**TABLE 1:** Primer sequences.

Gene symbol	Forward	Reverse
FAM134B	5′‐ GACAGCATCACAGTTTCAGGGAGA −3′	5′‐ AGACAGCCCATCCGTCTCCTT −3′
DERL2	5′‐CACCCAGAGTGACCTAAAGAAG‐3′	5′‐CCTGTTCCAGTGACGTGAGT‐3′
EDEM1	5′‐GAGCTGGGAGACTGGAAG‐3′	5′‐TCCCATGATGGTCTGTTCTG‐3′
SEL1L	5′‐TCAGCCACATGACCTGCAG‐3′	5′‐TCACAGGCTGGCACCATAG‐3′
HRD1	5′‐GCTTTGTCTTCCGCAGATG‐3′	5′‐GCCATCAGGTTGACTGCTC‐3′
LC3B	5′‐CGAGCTGGACAGTTTTTGCC‐3′	5′‐GCTGGCTGGTAGTTGTTGTCT‐3′
Beclin1	5′‐CGCGGCGCTCGAGATGTG‐3′	5′‐GTCGGTGCCACCTTTAGTTG‐3′
p62	5′‐AAAGGCTCTGGACAAAGTGG‐3′	5′‐GCCAGGTTTCATGTGTAGCG‐3′
GAPDH	5′‐CAATGACCCCTTCATTGACC‐3′	5′‐TTGATTTTGGAGGGATCTCG‐3′

### 2.9 Western blot

Protein extraction was performed on various cell lines and liver tissues. Subsequently, the proteins were separated by SDS‐PAGE and transferred onto PVDF membranes. After blocking with QuickBlockTM blocking buffer at room temperature for 20 min, the membranes were incubated overnight at 4°C with the primary antibody, followed by incubation with a secondary antibody (goat anti‐rabbit). The membranes were washed three times with TBST for 5 min each. Protein bands were visualized using an enhanced chemiluminescence (ECL) kit and analysed using a Vilber Fusion FX7 Spectra instrument. The protein expression levels were quantified using ImageJ software.

### 2.10 Immunohistochemistry

Paraffin‐embedded tissue sections were prepared by trimming and cutting 5 μm thick slices using a vibratome. After drying, deparaffinization and gradient alcohol hydration, antigen retrieval was conducted. The sections were incubated overnight at 37°C with primary antibodies against FAM134B, DERL2, EDEM1, SEL1L, HRD1 and GAPDH. After washing with Tris‐buffered saline, secondary antibodies were applied, followed by DAB staining and counterstaining with haematoxylin. Finally, the sections were examined under a microscope.

### 2.11 Statistical analysis

Statistical analysis of all data was carried out using GraphPad Prism 8.0 software. Descriptive statistics were employed to summarize general information. T‐tests were conducted to analyse quantitative data and compare means between two groups. Independent sample *t*‐tests were utilized to compare means between different groups. Additionally, one‐way anova was performed to compare means among multiple groups. The level of statistical significance was set at *p* < 0.05.

## 3 RESULTS

### 3.1 FAM134B expression is increased in human liver cancer tissues and cells

**FIGURE 1:**
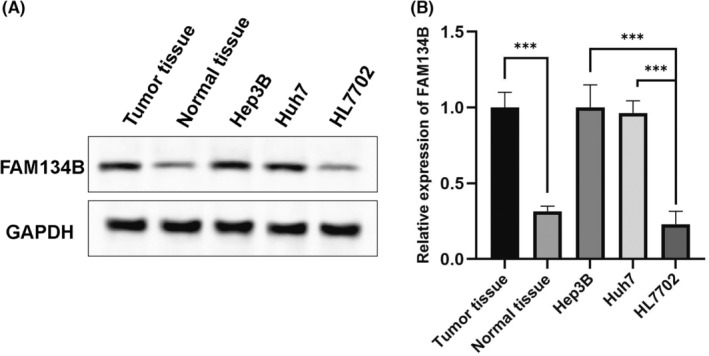
The upregulation of FAM134B expression in liver cancer. (A) The results of western blot analysis of FAM134B. (B) The quantitative analysis of band intensity. The statistical analysis revealed a significant increase in FAM134B expression in hepatocellular carcinoma, with a *p*‐value <0.001 (*** indicated).

Western blotting was employed to analyse the gene and protein expression levels of FAM134B in 10 pairs of adjacent non‐tumour and HCC tissues. The results revealed a significant increase in FAM134B expression in HCC tissues compared to adjacent non‐tumour tissues. Additionally, the evaluation of FAM134B expression in the human normal liver cell line HL7702 and HCC cell lines Hep3B and Huh7 demonstrated low levels of FAM134B expression in normal liver cells, whereas it was significantly upregulated in Hep3B and Huh7 HCC cell lines (*p* < 0.01, as shown in Figure 1A,B).

### 3.2 Stable cell lines with sh‐FAM134B interference were constructed by infecting Hep3B cells with lentivirus

**FIGURE 2:**
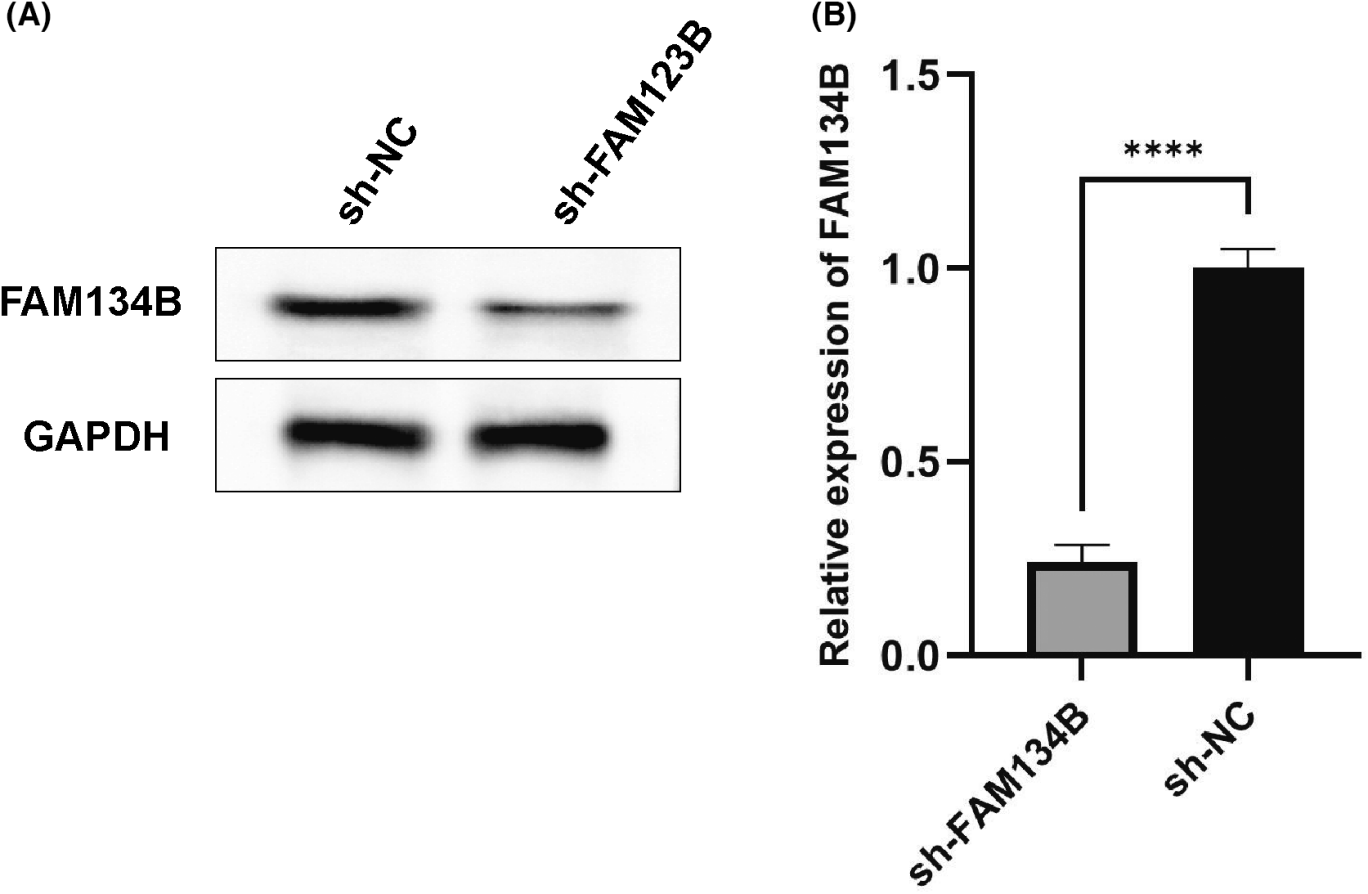
Generation of stable FAM134B knockdown cell lines via lentiviral transduction. (A) Western blot analysis of sh‐FAM134B. (B) Quantitative analysis of band intensity. **** indicates *p*‐value <0.0001.

Hep3B cells were genetically modified to have stable knockdown of FAM134B using lentiviral transduction of shRNA targeting FAM134B. The infection efficiency was assessed through RT‐qPCR and western blot to measure the transcriptional and protein levels of FAM134B, respectively. The results demonstrated a significant decrease in FAM134B expression in the sh‐FAM134B group compared to the sh‐NC group, confirming the successful generation of stable cell lines with FAM134B knockdown (*p* < 0.01, Figure 2A,B). These cell lines can be utilized for subsequent experimental studies.

### 3.3 sh‐FAM134B inhibits the proliferation and promotes apoptosis of HCC cells

**FIGURE 3:**
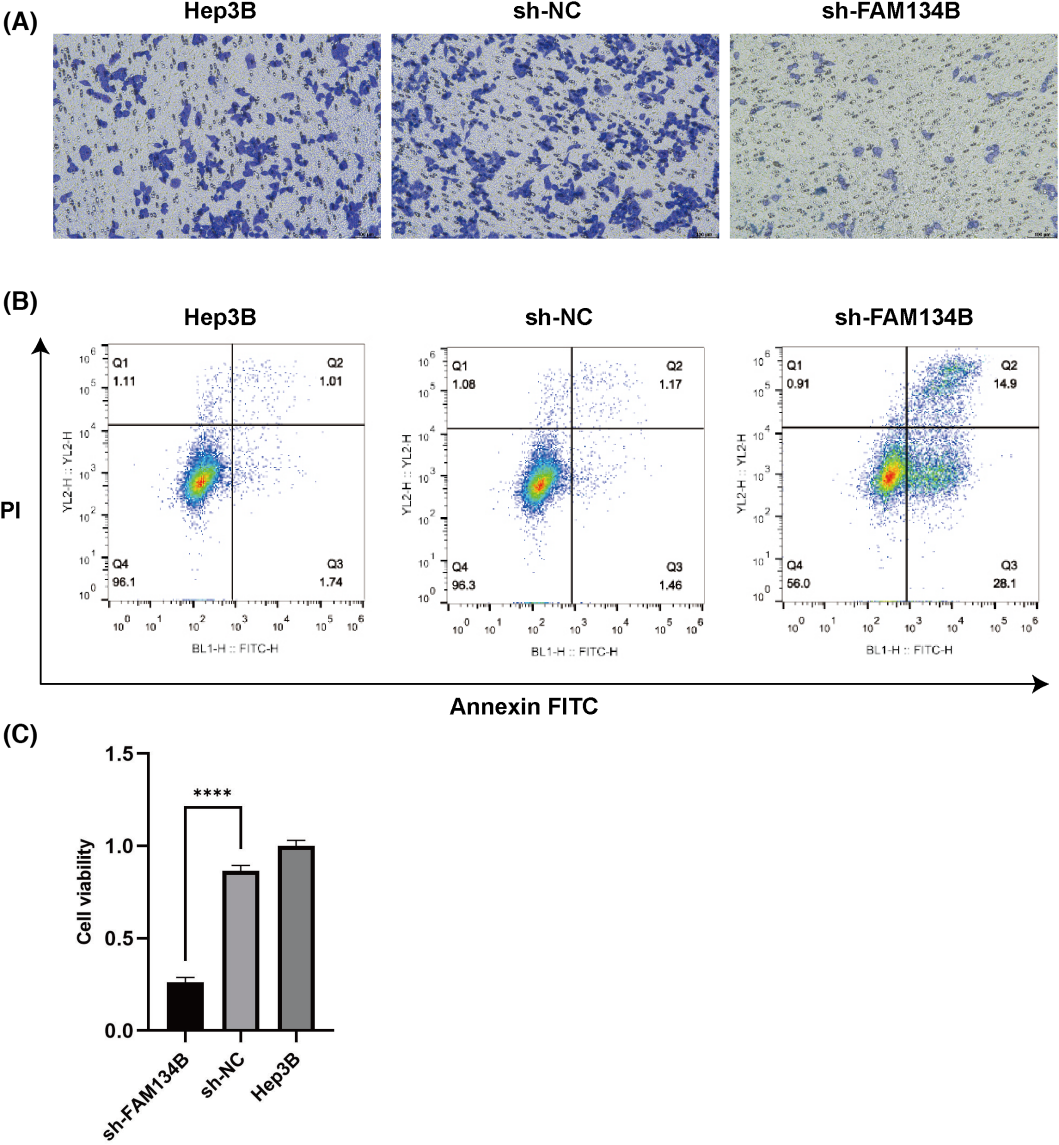
sh‐FAM134B suppresses HCC cell proliferation and promotes apoptosis. (A) Colony formation assay for different groups of cells. (B) Flow cytometry analysis of apoptotic cells. (C) CCK‐8 assay for cell viability. **** indicates *p*‐value <0.0001.

To investigate the impact of sh‐FAM134B on the proliferation of Hep3B, colony formation assays were conducted. The results exhibited a significant decrease in colony formation and inhibited cell growth in the sh‐FAM134B group when compared to the control group of Hep3B cells (Figure 3A). These findings suggest that knockdown of FAM134B expression can effectively suppresses HCC cell proliferation. Additionally, Annexin V/PI staining and flow cytometry analysis revealed a significant increase in apoptotic cells in the sh‐FAM134B group compared to the control group of Hep3B cells (Figure 3B). These results indicate that sh‐FAM134B prominently promote HCC cell apoptosis. Furthermore, cell viability assays revealed a significantly inhibition of cell viability in the sh‐FAM134B group when compared to the control group of Hep3B cells and the sh‐NC group, with statistically significant differences observed (*p* < 0.01, Figure 3C).

### 3.4 sh‐FAM134B promotes the formation of autophagosomes in HCC cells

**FIGURE 4:**
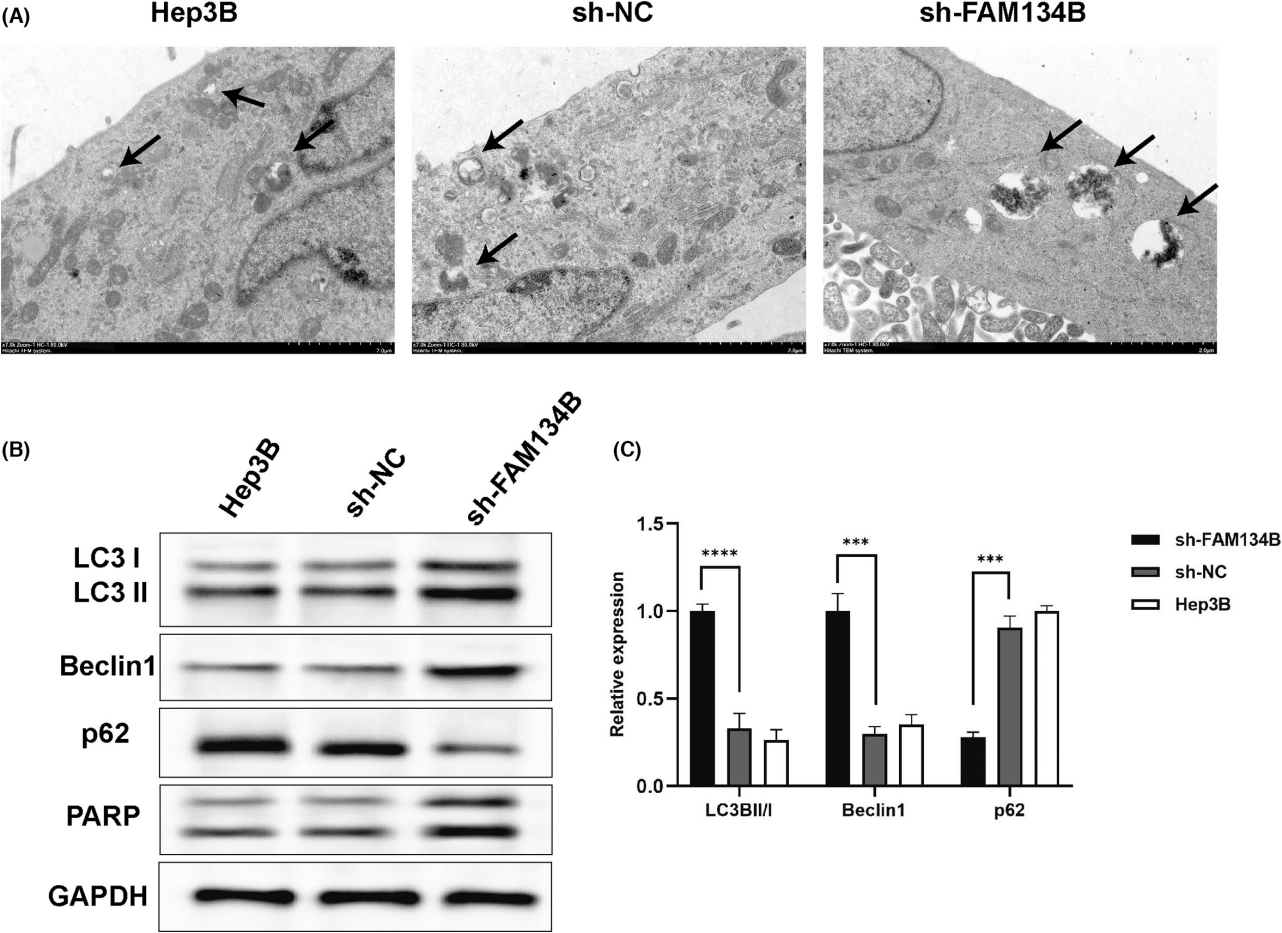
sh‐FAM134B promotes HCC cell autophagosome formation. (A) Transmission electron microscopy observation of autophagosome formation in different cell groups. (B) Expression bands of autophagy‐related proteins. (C) Protein quantification results. *** indicates *p*‐value <0.001; **** indicates *p*‐value <0.0001.

Transmission electron microscopy (TEM) analysis revealed a significant increase in the number of autophagosomes in the sh‐FAM134B group compared to the sh‐NC group (Figure 4A). To further investigate the process, RT‐qPCR and western blot analyses were conducted to assess the protein levels of autophagy‐related markers LC3B, Beclin1 and p62. The results demonstrated that knockdown of FAM134B expression in the sh‐FAM134B group led to an elevation in the LC3B‐II/I ratio, upregulation of Beclin1 protein levels and a decrease in p62 protein levels when compared to the control group of Hep3B cells and the sh‐NC group (*p* < 0.01, Figure 4B,C). These findings suggest that knockdown of FAM134B expression effectively induce autophagy in Hep3B HCC cells.

### 3.5 sh‐FAM134B promotes the expression of ER stress‐related factors in HCC cells

**FIGURE 5:**
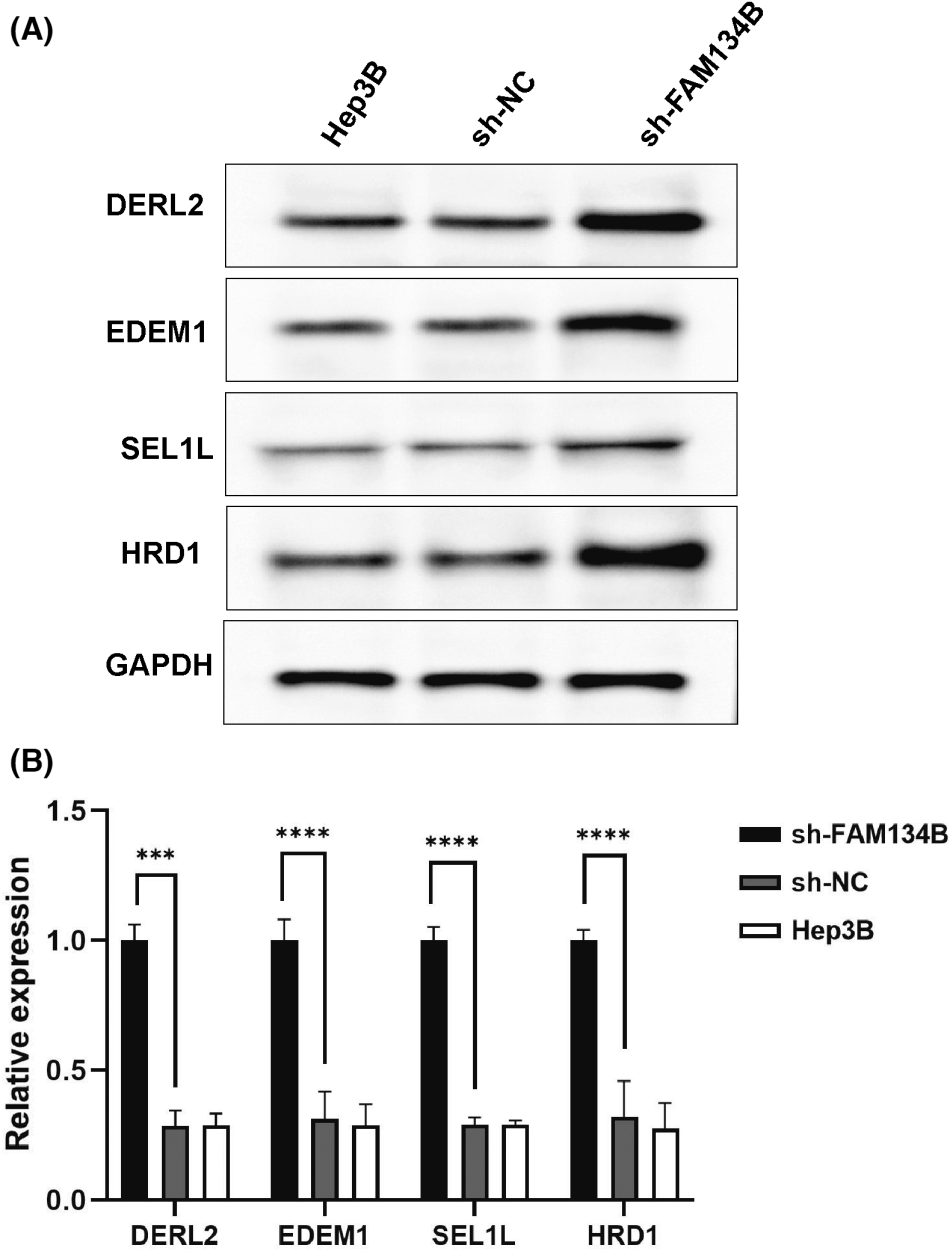
Illustrates the effects of sh‐FAM134B on the expression of ER stress‐related factors. (A) Shows the results of a western blot analysis of ER‐associated degradation (ERAD). (B) Provides a quantitative analysis of band intensity. The asterisks in the figure legend indicate the statistical significance of the results, with *** representing a *p*‐value <0.001 and **** representing a *p*‐value <0.0001. This information provides important insights into the mechanisms underlying ER stress in the context of our study.

To validate the capacity of sh‐FAM134B in inducing ER stress‐induced autophagy, Hep3B cells were transduced with FAM134B interference plasmids via lentiviral delivery and treated for 24 h. Total cellular proteins were extracted and subjected to western blot analysis to determine the expression of ER stress‐related factors. The results exhibited varying degrees of upregulation in the expression of DERL2, EDEM1, SEL1L and HRD1 in Hep3B cells of the sh‐FAM134B group when compared to the sh‐NC group (*p* < 0.05, Figure 5A,B), indicating the effective induction of ER stress in Hep3B liver cancer cells by sh‐FAM134B.

### 3.6 FAM134B and ERAD are abnormally increased in liver cancer tissues

**FIGURE 6:**
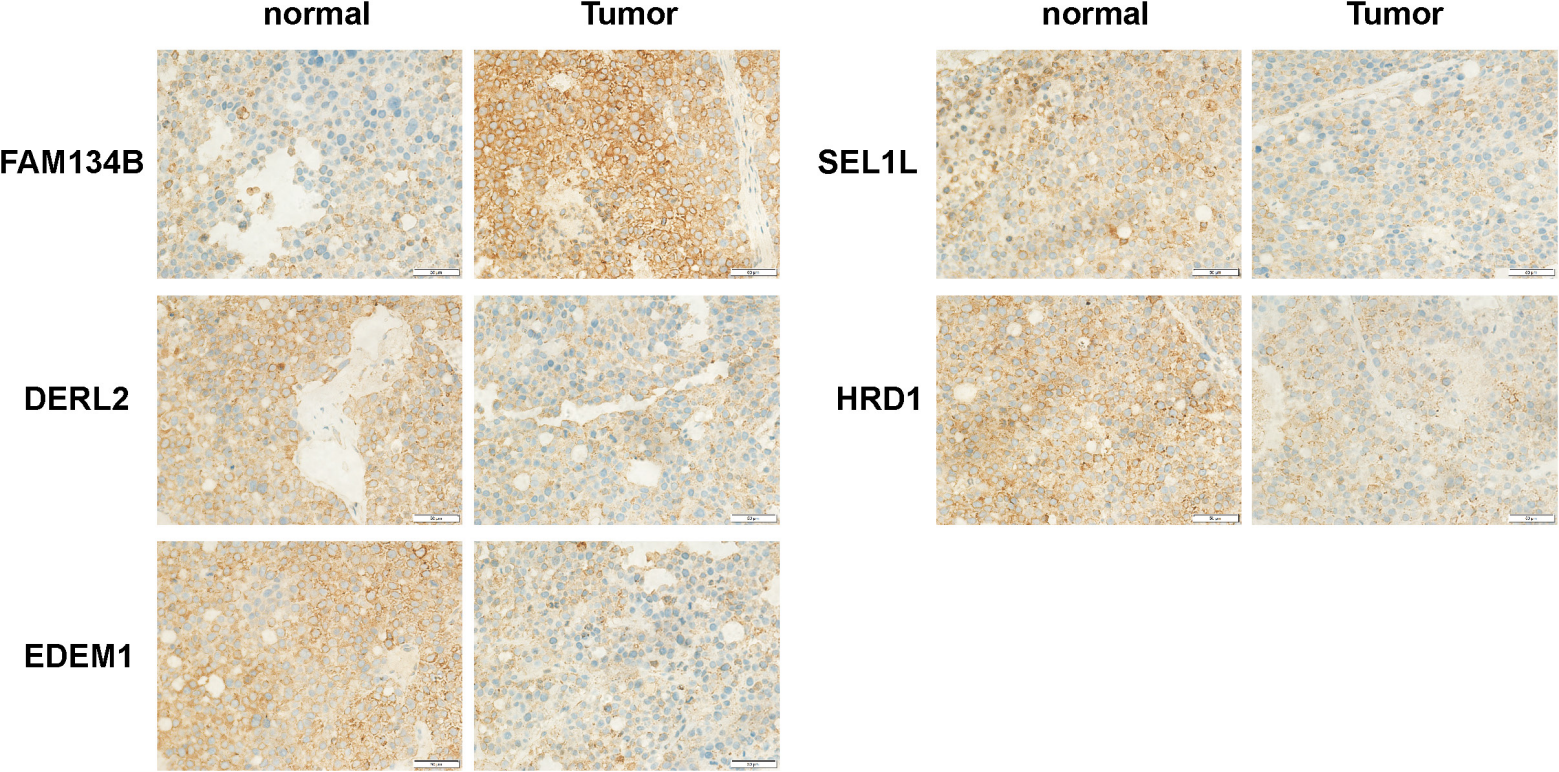
Immunohistochemical results show abnormal upregulation of FAM134B and ERAD expression in liver cancer tissues.

Subsequent immunohistochemical analyses were performed on HCC tissues and adjacent noncancerous tissues to explore the expression of endoplasmic reticulum (ER) stress‐related markers. The findings revealed a significant decrease in the number of positive cells for DERL2, EDEM1, SEL1L and HRD1 in HCC tissues, whereas the number of positive cells for FAM134B was significantly increased (Figure 6). These results suggest that FAM134B suppresses autophagy, promotes abnormal proliferation of HCC cells and facilitates the growth of liver cancer by specifically inhibiting the expression of ER stress‐related degradation factors like DERL2, EDEM1, SEL1L and HRD1.

## 4 DISCUSSION

The incidence of hepatocellular carcinoma (HCC) has exhibited a steady increase over the past 30 years.^17^ Currently, the primary treatment approaches for HCC encompass surgery, local ablation therapy, metastatic tumour therapy, chemotherapy, targeted therapy and immunotherapy.^18^ Targeted therapy predominantly involves the utilization of drugs that selectively target specific molecules in cancer cells, resulting in enhanced therapeutic efficacy. Numerous drugs in this domain are already available on the market or in the stage of research and development.^19^ Immunotherapy, on the contrary, represents a burgeoning field in liver cancer treatment, harnessing the power of the body's immune system to combat liver cancer cells, with progressively recognized efficacy and safety.^20^ Clinical studies have shown that following surgical resection of liver cancer, the recurrence rate within 3 years can be as high as approximately 50% and over 70% within 5 years.^21^ To minimize the recurrence rate of liver cancer, it is imperative to gain a deeper understanding of the biological mechanisms of liver cancer, improve the early detection rate and adopt a comprehensive approach incorporating various treatment modalities.

This study revealed a significant upregulation of FAM134B in human liver cancer tissues as well as HCC cell lines Hep3B and Huh7. Interference with FAM134B expression effectively inhibited Hep3B cell proliferation. In addition, sh‐FAM134B substantially promoted HCC cell apoptosis and effectively suppressed the migration and invasion of HCC cells, including Hep3B cells. FAM134B, residing as a transmembrane protein on the endoplasmic reticulum (ER) membrane, participates in diverse biological processes such as cell apoptosis and stress response under specific conditions. Previous studies have demonstrated that reducing FAM134B expression in liver cancer cell models can attenuate various biological behaviours of liver cancer, including migration, invasion and metastasis.^22^ Furthermore, employing small interfering RNA (siRNA) technology to diminish FAM134B expression increases the oxidative stress sensitivity in liver cancer cells, ultimately preventing their growth and dissemination.^23^ These research findings underscore the potential significance of the FAM134B gene in the initiation and progression of liver cell cancer and propose it as a prospective target for therapeutic intervention. However, given the limited research available to ascertain the precise mechanisms by which FAM134B operates in the biological processes of liver cell cancer, further investigations are necessary to unravel its relationship with liver cell cancer.

In addition, this study demonstrated that sh‐FAM134B effectively induce autophagy in Hep3B liver cancer cells while promoting endoplasmic reticulum stress. FAM134B promotes the growth of liver cancer cells through its involvement in the endoplasmic reticulum stress pathway.^24^ Endoplasmic reticulum stress plays an important role in liver growth and regenerative processes, particularly in the activation and proliferation of liver cancer cells.^25^ Currently, research has revealed that endoplasmic reticulum stress may also inhibit cell autophagy, leading to metabolic disorders, apoptosis, and exacerbated inflammation.^26^ The process of cell autophagy can be regulated by endoplasmic reticulum stress through various signalling pathways such as ATF4 and CHOP, thereby maintaining cellular homeostasis.^27^ Simultaneously, cell autophagy facilitates the clearance of non‐degradable proteins and endoplasmic reticulum aggregates, thereby mitigating the intensity and duration of endoplasmic reticulum stress.

The interplay between the endoplasmic reticulum stress pathway and cell autophagy has significant implications for the growth, metabolism and metastasis of liver cancer cells. Endoplasmic reticulum stress can stimulate the autophagy in liver cancer cells, facilitating the clearance of damaged substances such as misfolded proteins and organelles by modulating autophagy signalling pathways. This, in turn, reduces endoplasmic reticulum stress in liver cancer cells, enabling their continued growth and proliferation.^28^ Conversely, endoplasmic reticulum stress can suppress autophagy and promote apoptosis in liver cancer cells by modulating the autophagy pathway.^29^ Studies have revealed that endoplasmic reticulum stress can inhibit the expression of autophagy‐related genes, including ATG12 and ATG5, allowing liver cancer cells to evade autophagic degradation and enhance their capacity for growth and proliferation. Consequently, the role of autophagy in liver cancer cells is counteracted by endoplasmic reticulum stress.^30^ In conclusion, the interplay between ER stress and cell autophagy plays a crucial role in the progression of liver cancer. Hence, future investigation should focus on elucidating the underlying mechanisms of this interaction to pave the way for novel therapeutic strategies in liver cancer.

In conclusion, the inhibitory effect of FAM134B on hepatocellular carcinoma (HCC) cell autophagy is attributed to its suppression of endoplasmic reticulum stress‐related degradation factors, including DERL2, EDEM1, SEL1L and HRD1. Consequently, the dysregulation of FAM134B expression promotes the progression of liver cancer. Notably, the level of FAM134B expression can serve as an indicator of endoplasmic reticulum stress and potentially function as a biomarker for liver cancer. Hence, exploring the role of FAM134B in liver cancer holds significant promise for future research in the field of liver cancer treatment.

## AUTHOR CONTRIBUTIONS

**Heng Li:** Writing – review and editing (equal). **Houhong Wang:** Data curation (equal). **Lu Liu:** Writing – original draft (equal). **Huihui Gong:** Visualization (equal).

## CONFLICT OF INTEREST STATEMENT

The authors declare that they have no competing interests.

## CONSENT FOR PUBLICATION

All authors consent to publication.

## Data Availability

The data that support the findings of this study are available on request from the corresponding author. The data are not publicly available due to privacy or ethical restrictions.
